# Association Between Lifestyle Factors and the Prevalence of Non-Communicable Diseases in Saudi Adults Across Different Age Groups: A Cross-Sectional Study

**DOI:** 10.3390/diseases14050163

**Published:** 2026-05-07

**Authors:** Somia A. Nassar

**Affiliations:** Department of Medical Laboratory Sciences, College of Applied Medical Sciences, Prince Sattam bin Abdulaziz University, Alkharj 11942, Saudi Arabia; s.salem@psau.edu.sa; Tel.: +966-500-938-476

**Keywords:** non-communicable diseases (NCDs), physical activity (PA), diet, obesity, smoking, diabetes mellitus, hypertension, osteoporosis (OP), chronic obstructive pulmonary disease (COPD), aging diseases

## Abstract

Objectives: This cross-sectional study examined associations of lifestyle factors (physical activity (PA), diet, obesity, and smoking), age groups, and sex with the prevalence of non-communicable diseases (NCDs) in Saudi adults, including the World Health Organization (WHO) core NCDs (type 2 diabetes (T2DM), hypertension (HTN), chronic obstructive pulmonary disease (COPD) and NCD-associated conditions (osteoporosis (OP), chronic kidney disease (CKD)). Methods: A cross-sectional study was conducted across Saudi Arabia involving 2877 participants aged ≥30 years. Data were collected via an electronic survey using a standardized questionnaire. PA was assessed using the International PA Questionnaire (IPAQ-SF), diet using the Alternative Healthy Eating Index (AHEI), and smoking using the WHO Global Adult Tobacco Survey. Results: Prevalence estimates were: OP 22%, diabetes 21.8%, HTN 13.4%, COPD 4.3%, and CKD 5.1%. All conditions were more prevalent among inactive vs. active individuals (e.g., diabetes: 23.5% vs. 18.8%). An unhealthy diet was associated with higher prevalence (e.g., HTN: 16.3% vs. 10.8%). Obesity showed the strongest association with diabetes (37.1% in obese vs. 14.9% in normal-weight). Smoking was associated with higher prevalence (e.g., COPD: 7.9% vs. 3.7%). Women had higher prevalence than men for most conditions (e.g., OP: 23.4% vs. 19.7%), except COPD (5.1% in men vs. 3.8% in women). Prevalence increased with age (e.g., HTN: 7.2% at age 30–40 vs. 17.3% at age > 60). All comparisons were tested using chi-square tests (*p* < 0.05). Conclusions: The findings underscore an urgent need for targeted public health interventions to promote PA, improve nutrition, combat obesity, and reduce smoking.

## 1. Introduction

Advances in social, economic, and scientific fields have been associated with reduced age-related mortality, accelerating global population aging [1]. The Kingdom of Saudi Arabia (KSA) mirrors this trend: a 30-year increase in life expectancy since 1960, reaching 76.4 years overall in 2021, attributed to improved healthcare and living conditions [2].

However, a critical research gap exists: while individual lifestyle factors (physical inactivity, poor diet, smoking, and obesity) are known to be associated with age-related diseases, no study has comprehensively examined the associations of these multiple lifestyle factors with multi-morbidity among Saudi Arabia’s rapidly growing older adult population. Population-level, context-specific data from the Arabian Gulf region addressing this gap are scarce.

Therefore, this study’s objective is to examine the associations of individual lifestyle factors with prevalent age-related morbidities in a large Saudi cohort, providing evidence to guide culturally appropriate preventive measures.

The WHO identifies four core NCD groups: cardiovascular diseases (CVDs), cancers, chronic respiratory diseases, and T2DM [3]. Accordingly, we classify T2DM, COPD, and HTN (a CVD and major risk factor [4]) as WHO core NCDs. CKD and OP are not WHO core NCDs. However, both are highly prevalent among older Saudis, share the same lifestyle risk factors, and contribute substantially to morbidity. CKD commonly complicates T2DM and HTN, while OP increases fracture risk. We therefore include them as NCD-associated conditions for a comprehensive assessment.

Aging involves physiological decline, which is associated with increased vulnerability to chronic disease [5]. Saudi Arabia’s elderly population aged 65 years and above is projected to continue increasing, reaching 18.4% of the total population in 2050, accompanied by rising NCD prevalence, including HTN, CVD, T2DM, OP, obesity, asthma, kidney disorders, cancer, and metabolic syndrome [6]. A recent cross-sectional study reported that among Saudi adults aged 65 years and older, the prevalence of hypertension was 59.6%, diabetes was 52.8%, and high cholesterol was 49.5% [7].

Lifestyle factors—including PA, diet, smoking, and body mass—are established determinants of chronic disease development, morbidity, and mortality [8]. In KSA, adults aged 30–70 face an 18.6% probability of premature death from NCDs such as ischemic heart disease, cancer, T2DM, and chronic respiratory diseases [9]. Higher levels of PA are associated with lower prevalence of age-related diseases and better quality of life in aging populations. Conversely, physical inactivity is a major public health concern, particularly among older adults [6]. In KSA, rapid socioeconomic and urban development has been accompanied by shifts in lifestyle patterns [10]; 66.6% of the population is physically inactive, with the highest rates in the 55–64 age group [6]. In 2019, insufficient PA was associated with 4.8% of deaths and 2.6% of NCD-related disability-adjusted life-years nationally [11].

In KSA, a multi-city study found that 34.4% of participants did not follow a healthy diet [12], while only 1.53% met all Ministry of Health food group recommendations and 43.2% met none. This poor dietary compliance is associated with Saudi Arabia’s high NCD prevalence, which accounts for 73% of national mortality [13].

Cigarette smoke is a well-established correlate of CVD, as well as respiratory, and other systemic diseases [14]. In KSA, smoking prevalence ranges from 17.3% (always smoking) to 30.7% (active smokers)—with higher rates among men—and is identified as the most common lifestyle-related factor associated with NCDs [15]. A study in eastern Saudi Arabia identified smoking as the leading CVD correlate (9.4%) [16], alongside HTN (40.8%) and T2DM (39.6%) [17].

KSA ranks 14th globally in obesity prevalence at 35.4% [18], though a recent survey reported 25.3% obesity and 48.1% overweight in the Aseer region [19]. Higher body mass index (BMI) is associated with greater prevalence of NCDs, including T2DM (OR = 1.52), hypercholesterolemia (OR = 1.69), HTN (OR = 1.61), lung diseases (OR = 1.69), rheumatoid arthritis (OR = 1.57), sleep apnea (OR = 1.82), colon diseases (OR = 1.31), and thyroid disorders (OR = 1.8) [12].

While individual associations between lifestyle factors (physical inactivity, unhealthy diet, smoking, and obesity) and age-related NCDs are well established, this investigation directly addresses the identified gap by examining these associations in a large Saudi cohort.

## 2. Materials and Methods

### 2.1. Study Design and Data Collection

This cross-sectional study recruited 2877 Saudi citizens aged ≥30 years across multiple provinces in KSA using convenience sampling.

Data were collected via a Google Forms questionnaire disseminated through social media (Facebook, WhatsApp, Twitter, Telegram), targeted email campaigns to institutional employees, and in-person interviews at social and workplace settings. Participants were encouraged to share the survey within their networks. Sociodemographic characteristics (sex, marital status, education, occupation) were recorded.

Participation was voluntary, anonymous, and confidential. Non-residents, individuals living outside KSA, those under 30, and incomplete responses were excluded.

### 2.2. Questionnaire

This online questionnaire assessed lifestyle habits and age-related diseases among individuals aged ≥30 years in Saudi Arabia, including both genders. Prior to administration, a pilot study with 25 participants aged ≥30 was conducted to: evaluate question clarity and understandability, estimate completion time, identify potential issues or ambiguities, and ensure efficient questionnaire administration. Based on pilot feedback, the questionnaire was refined to improve clarity and comprehension, with specific questions and answer options modified. The final Arabic version consisted of six sections and took approximately 10–15 min to complete. Participants received informed consent detailing the study’s objectives, inclusion criteria, data confidentiality, and estimated completion time prior to beginning.

The questionnaire comprised six sections: the first section collected sociodemographic data (age, gender, nationality, marital status, employment, education); the second section assessed PA using a modified Arabic IPAQ Short Form (IPAQ-SF), which evaluated leisure-time activities (e.g., walking, cycling, swimming) by intensity, frequency, and duration over the previous seven days to categorize participants into activity levels (sedentary, low, moderate, or high). The third section assessed dietary habits, including portion sizes, food frequency and diversity, consumption of specific food groups (fruits, vegetables, whole grains, dairy, meat, processed foods), snacking, and emotional eating. The fourth section addressed anthropometric measurements and weight management behaviors, collecting self-reported weight and height for BMI calculation, and inquiring about weight loss willingness, dietary change intentions, and PA engagement. The fifth section assessed smoking habits (current, former, never smokers), including tobacco type, intensity (cigarettes/day), and duration for current smokers. The sixth section evaluated medical status, including diagnoses of T2DM, HTN, respiratory/renal conditions and OP; frequency of recent blood sugar/Blood pressure (BP) measurements; medication use; musculoskeletal conditions (joint pain, fractures, limited movement, bone mineral density (BMD) measurement); and symptoms of respiratory/urinary conditions (cough, shortness of breath, dysuria, polyuria, urolithiasis).

### 2.3. Study Variables

#### 2.3.1. Physical Activity (PA)

PA was assessed using the short form of the IPAQ, which was developed by an International Consensus Group spearheaded by the WHO. The IPAQ has demonstrated acceptable test-retest reliability and moderate correlation with objective activity measures in adult populations. Participants reported the frequency (days per week) and duration (minutes per day) of walking, moderate-intensity, and vigorous-intensity activity. Participants were classified as physically active if they engaged in at least 150 min of moderate-intensity activity, or 75 min of vigorous-intensity activity, or an equivalent combination per week. Those not meeting this threshold were classified as physically inactive. This binary classification aligns with public health guidelines defining a minimum threshold for health benefits, facilitates straightforward interpretation, enables comparison with population-level surveillance data (e.g., WHO Global Action Plan on PA) [20], and directly identifies individuals failing to meet recommended activity levels—the primary target for lifestyle interventions.

#### 2.3.2. Diet

Dietary habits were assessed using a 14-item food frequency questionnaire capturing portion sizes, food frequency, and diversity across key food groups (fruits, vegetables, whole grains, dairy, meat, processed foods, snacks, and emotional eating behaviors). Responses were converted into daily or weekly consumption estimates. A diet quality score was calculated based on the AHEI, a validated instrument for assessing dietary quality. Each of the 11 components was scored from 0 to 10, yielding a total score range of 0–110.

Participants with a total AHEI score of ≥65 were classified as adhering to a healthy diet, while those scoring <65 were classified as following an unhealthy diet. This binary classification is grounded in WHO dietary recommendations, aligns with global public health targets, facilitates comparison with national and international surveillance data (e.g., the WHO Global Action Plan for the Prevention and Control of NCDs) [3], and directly identifies individuals failing to meet recommended dietary guidelines—a key modifiable risk factor for chronic diseases.

#### 2.3.3. Smoking

Tobacco use was assessed using standardized questions adapted from the WHO GATS. Participants were asked: “Do you currently smoke any tobacco products, including cigarettes, waterpipe (shisha), cigars, or pipes, on a daily basis, less than daily, or not at all?”

Participants who responded “daily” were classified as smokers. Those who responded “less than daily” or “not at all” were classified as non-smokers. Former smokers (those who had smoked ≥100 tobacco products in their lifetime but did not currently smoke) were included in the non-smoker group unless otherwise analyzed separately.

This binary classification captures habitual daily tobacco exposure—the primary driver of smoking-related health risks. It aligns with standard public health surveillance definitions (e.g., the WHO STEPwise approach and GATS) [21], facilitates straightforward interpretation, enables comparison with national and international prevalence data, and directly identifies individuals at elevated risk for tobacco-related diseases.

#### 2.3.4. Obesity

Classifying participants based on WHO BMI cutoffs (normal weight < 25 kg/m^2^; overweight ≥ 25 to <30 kg/m^2^; obese ≥ 30 kg/m^2^) provides a standardized, internationally recognized measure of obesity. This categorization aligns with global public health surveillance frameworks (e.g., WHO Obesity and Overweight Fact Sheet) [22], enables direct comparison with national and international prevalence data, and facilitates clinical interpretation given the well-established dose-dependent relationship between elevated BMI and chronic disease risk. By distinguishing normal weight, overweight, and obesity, this classification identifies subgroups at progressively higher risk for CVD, T2DM, and other obesity-related conditions, thereby informing targeted preventive and therapeutic interventions.

#### 2.3.5. Data Quality Control

Participants with implausible BMI values (<12 or >60 kg/m^2^) or unrealistic PA reports (>16 h/day) were excluded from relevant analyses to reduce misclassification bias.

### 2.4. Classification of Outcome Variables

The WHO identifies four core NCD groups: CVD, cancers, chronic respiratory diseases, and T2DM [3]. We classify T2DM, COPD, and HTN (a CVD [4]) as WHO core NCDs.

CKD and OP are not WHO core NCDs. However, both are highly prevalent among older Saudis, share the same lifestyle risk factors, and contribute substantially to morbidity. CKD commonly complicates T2DM and HTN, while OP increases fracture risk. We therefore include them as NCD-associated conditions.

### 2.5. Statistical Analysis

All statistical analyses were performed using Microsoft Excel (version 16.0). Associations between categorical variables were assessed using the chi-square test. Descriptive statistics (prevalence percentages) were calculated for each NCD across lifestyle factor categories (PA, diet, obesity, smoking), age groups, and sex.

Chi-square tests were used to compare disease prevalence across: PA (active vs. inactive), diet (healthy vs. unhealthy), smoking (smoker vs. non-smoker), obesity (normal weight, overweight, obese), age groups (30–40, 41–50, 51–60, >60 years) and sex (male vs. female)

For 2 × 2 comparisons, degrees of freedom (df) = 1. For obesity (3 categories), df = 2. For age groups (4 categories), df = 3. Statistical significance was set at *p* < 0.05.

## 3. Results

### 3.1. Lifestyle Profiles of the Study Participants

The study found that 36.3% of participants were physically active versus 63.7% inactive; 17.1% were smokers and 82.9% non-smokers. Body weight distribution showed 45.9% as normal weight, 31.4% overweight and 22.7% obese. Regarding diet, 53.3% adhered to a healthy diet while 46.7% followed an unhealthy diet (Table 1).

### 3.2. Classification of Outcome Variables

In this study, we include both WHO core NCDs (T2DM, COPD, and HTN) and NCD-associated conditions (OP and CKD), which share common lifestyle risk factors [3,4]. The classification of each condition is indicated in Table 2.

### 3.3. Prevalence of Age-Related Diseases by Age, Sex, and Lifestyle Factors

The prevalence of T2DM, HTN, OP, respiratory diseases, and renal diseases is presented overall and by age, sex, PA, diet, obesity, and smoking. OP was the most common condition (22.0%), followed by T2DM (21.8%). Disease prevalence was associated with age and was consistently higher among physically inactive participants, those with unhealthy diets, and smokers. While obesity was associated with substantially higher prevalence of T2DM, HTN, respiratory diseases, and renal diseases, this gradient was not evident for OP (Table 3).

### 3.4. Statistical Associations Between Lifestyle Factors, Age Groups, Sex, and NCDs (Chi-Square Tests)

Table 4 summarizes the chi-square test results for all associations between lifestyle factors, age groups, sex, and NCDs.

PA was significantly associated with T2DM (χ^2^ = 8.81, *p* = 0.003), HTN (χ^2^ = 7.41, *p* = 0.007), and OP (χ^2^ = 8.92, *p* = 0.003), but not with respiratory (*p* = 0.119) or renal conditions (*p* = 0.427). Thus, physical inactivity was significantly associated with higher prevalence of T2DM, HTN, and OP compared to active individuals.

Diet was significantly associated with T2DM (χ^2^ = 11.36, *p* = 0.001), HTN (χ^2^ = 18.80, *p* < 0.001), and respiratory conditions (χ^2^ = 9.55, *p* = 0.002), but not with OP (*p* = 0.791) or renal conditions (*p* = 0.718). Thus, an unhealthy diet was associated with higher prevalence of T2DM, HTN, and respiratory diseases.

Smoking was significantly associated with T2DM (χ^2^ = 6.20, *p* = 0.013), respiratory conditions (χ^2^ = 19.57, *p* < 0.001), and renal conditions (χ^2^ = 4.57, *p* = 0.033), but not with HTN (*p* = 0.215) or OP (*p* = 0.242). Thus, smoking was associated with higher prevalence of T2DM, respiratory diseases, and renal diseases.

Obesity was significantly associated with all conditions except OP (*p* = 0.905) and renal conditions (*p* = 0.143). The strongest association was observed for T2DM (χ^2^ = 136.47, *p* < 0.001), where higher BMI categories were associated with progressively higher prevalence (14.9% in normal weight, 20.8% in overweight, and 37.1% in obese). Obesity was also significantly associated with HTN (χ^2^ = 33.95, *p* < 0.001) and respiratory conditions (χ^2^ = 21.42, *p* < 0.001). No significant associations were observed for OP (*p* = 0.905) or renal conditions (*p* = 0.143).

Age groups were significantly associated with all conditions except respiratory conditions (*p* = 0.058). The strongest age-related associations were observed for OP (χ^2^ = 148.63, *p* < 0.001) and HTN (χ^2^ = 35.99, *p* < 0.001).

Sex was significantly associated only with OP (χ^2^ = 5.01, *p* = 0.025), where females had a higher prevalence (23.4%) compared to males (19.7%). No significant sex differences were observed for T2DM (*p* = 0.374), HTN (*p* = 0.174), or renal conditions (*p* = 0.740). Respiratory conditions showed a borderline non-significant association (*p* = 0.052), with higher prevalence in males (5.1% vs. 3.8%).

### 3.5. Type 2 Diabetes Mellitus

**Figure 1:**
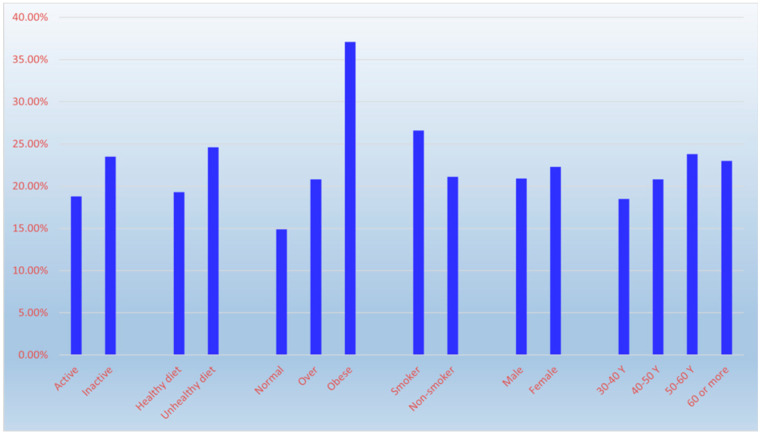
The influence of age, sex and lifestyle factors on diabetes mellitus prevalence.

The study found an overall T2DM prevalence of 21.8%, increasing with age: 18.5% (30–40 years), 20.8% (41–50), 23.8% (51–60), and 23.0% (>60) (χ^2^ = 8.47, *p* = 0.037). Prevalence was higher in females (22.3%) than males (20.9%), but this difference was not statistically significant (χ^2^ = 0.79, *p* = 0.374). Prevalence was significantly lower among physically active (18.8%) vs. inactive (23.5%) individuals (χ^2^ = 8.81, *p* = 0.003), and among those with healthy diets (19.3%) vs. unhealthy diets (24.6%) (χ^2^ = 11.36, *p* = 0.001). Obesity was significantly associated with higher prevalence (37.1% in obese, 20.8% in overweight, 14.9% in normal-weight) (χ^2^ = 136.47, *p* < 0.001). Smoking was also significantly associated with higher prevalence (26.6% in smokers vs. 21.1% in non-smokers) (χ^2^ = 6.20, *p* = 0.013) (Figure 1 and Table 3).

### 3.6. Hypertension

**Figure 2:**
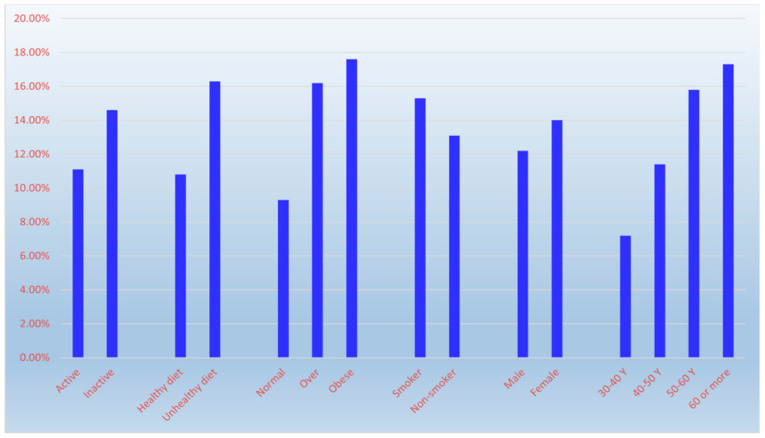
The influence of age, sex and lifestyle factors on hypertension prevalence.

The study found an overall HTN prevalence of 13.4%, associated with age: 7.2% (30–40 years), 11.4% (41–50), 15.8% (51–60), and 17.3% (>60) (χ^2^ = 35.99, *p* < 0.001). Prevalence was higher in women (14.0%) than men (12.2%), but this difference was not statistically significant (χ^2^ = 1.85, *p* = 0.174). Prevalence was significantly lower among physically active (11.1%) vs. inactive (14.6%) individuals (χ^2^ = 7.41, *p* = 0.007), and among those with healthy diets (10.8%) vs. unhealthy diets (16.3%) (χ^2^ = 18.80, *p* < 0.001). Obesity was significantly associated with higher prevalence (17.6% in obese, 16.2% in overweight, 9.3% in normal-weight) (χ^2^ = 33.95, *p* < 0.001). Smoking was associated with higher prevalence (15.3% in smokers vs. 13.1% in non-smokers), but this difference was not statistically significant (χ^2^ = 1.54, *p* = 0.215) (Figure 2 and Table 3).

### 3.7. Osteoporosis

**Figure 3:**
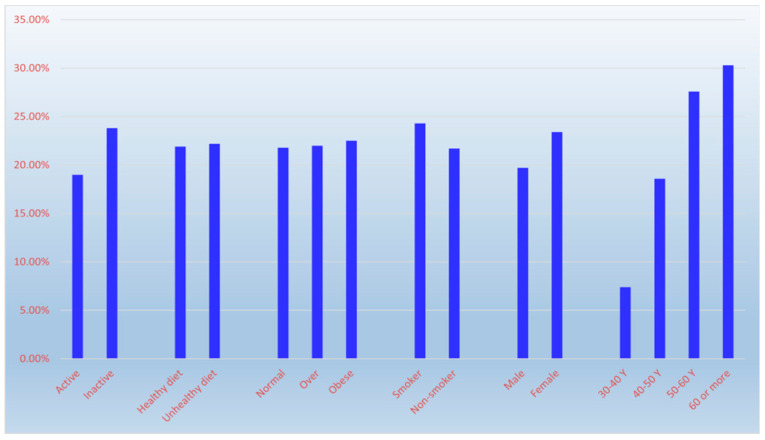
The influence of age, sex and lifestyle factors on osteoporosis prevalence.

The study revealed an overall OP prevalence of 22%, which was significantly associated with age: 7.4% (30–40 years), 18.6% (41–50), 27.6% (51–60), and 30.3% (>60) (χ^2^ = 148.63, *p* < 0.001). Prevalence was significantly higher in females (23.4%) than males (19.7%) (χ^2^ = 5.01, *p* = 0.025). Prevalence was significantly lower among physically active (19.0%) vs. inactive (23.8%) individuals (χ^2^ = 8.92, *p* = 0.003). The difference by diet was minimal (21.9% healthy diet vs. 22.2% unhealthy diet) and not statistically significant (χ^2^ = 0.07, *p* = 0.791). No significant association was observed with body weight (21.8% normal, 22.0% overweight, 22.5% obese) (χ^2^ = 0.20, *p* = 0.905). Smoking was associated with slightly higher prevalence (24.3% smokers vs. 21.7% non-smokers), but this difference was not statistically significant (χ^2^ = 1.37, *p* = 0.242) (Figure 3 and Table 3).

### 3.8. Respiratory Conditions

**Figure 4:**
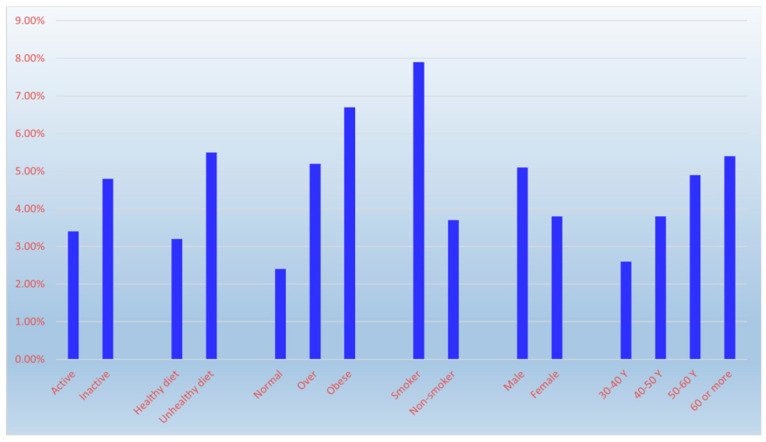
The influence of age, sex and lifestyle factors on chronic obstructive pulmonary disease prevalence.

Respiratory conditions had an overall prevalence of 4.3%, with prevalence varying by age —2.6% (30–40 years), 3.8% (41–50), 4.9% (51–60), and 5.4% (>60)—but this association was not statistically significant (χ^2^ = 7.47, *p* = 0.058). Prevalence was higher in males (5.1%) than females (3.8%), but this difference was not statistically significant (χ^2^ = 3.78, *p* = 0.052, borderline). Prevalence was lower among physically active (3.4%) vs. inactive (4.8%) individuals, but this difference was not statistically significant (χ^2^ = 2.43, *p* = 0.119). Prevalence was significantly lower among those with healthy diets (3.2%) vs. unhealthy diets (5.5%) (χ^2^ = 9.55, *p* = 0.002). Obesity was significantly associated with higher prevalence (6.7% obese, 5.2% overweight, 2.4% normal-weight) (χ^2^ = 21.42, *p* < 0.001). Smoking was significantly associated with higher prevalence (7.9% smokers vs. 3.7% non-smokers) (χ^2^ = 19.57, *p* < 0.001) (Figure 4 and Table 3).

### 3.9. Chronic Kidney Disease

**Figure 5:**
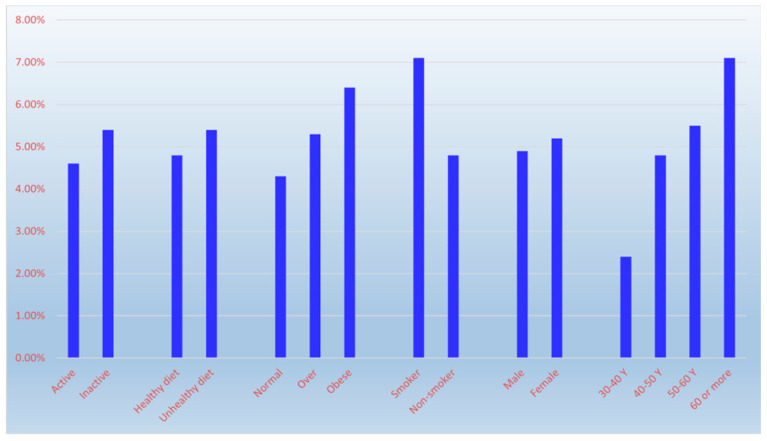
The influence of age, sex and lifestyle factors on chronic kidney disease prevalence.

The overall prevalence of renal conditions was 5.1%, significantly associated with age from 2.4% (30–40 years) to 7.1% (>60 years) (χ^2^ = 12.31, *p* = 0.006). Prevalence was slightly higher in females (5.2%) than males (4.9%), but this difference was not statistically significant (χ^2^ = 0.11, *p* = 0.740). Prevalence was higher among physically inactive (5.4% vs. 4.6%) people and those with unhealthy diets (5.4% vs. 4.8%), but these differences were not statistically significant (*p* = 0.427 and *p* = 0.718, respectively). Prevalence varied by body weight (4.3% normal, 5.3% overweight, 6.4% obese), but this association was not statistically significant (χ^2^ = 3.89, *p* = 0.143). Smoking was significantly associated with higher prevalence (7.1% smokers vs. 4.8% non-smokers) (χ^2^ = 4.57, *p* = 0.033) (Figure 5 and Table 3).

## 4. Discussion

### 4.1. Type 2 Diabetes Mellitus (T2DM)

In this cohort, the overall prevalence of T2DM was 21.8%, which aligns closely with the nationally reported adult prevalence of 23.7% [23], suggesting our sample is representative of the broader Saudi population with respect to T2DM burden.

T2DM prevalence increased progressively from 18.5% (age 30–40) to 23.8% (age 51–60) (χ^2^ = 8.47, *p* = 0.037). This age-related increase is consistent with well-established age-related physiological changes including insulin resistance and declining pancreatic function, and prevalent vitamin D deficiency in KSA’s elderly also impairs insulin sensitivity [6,24]. However, we observed a plateau above age 60 (23.0%), which may reflect survivor bias or improved disease management among older adults who have survived with T2DM. This plateau warrants further investigation in longitudinal Saudi cohorts.

Contrary to previous Saudi studies reporting substantially higher T2DM prevalence among women attributed to greater obesity rates (59.2% vs. 40.8%) [25,26], we found a modest difference (22.3% in females vs. 20.9% in males) that was not statistically significant (χ^2^ = 0.79, *p* = 0.374). This narrower gap suggests that sex disparities in T2DM may be diminishing in KSA, possibly due to changing lifestyle patterns or improved healthcare access for women.

Inactive participants had significantly higher T2DM prevalence (23.5%) than active participants (18.8%) (χ^2^ = 8.81, *p* = 0.003)—a 4.7 percentage point difference. While this association is expected [27], the magnitude in our cohort is smaller than reported in some Western populations [28,29], potentially due to residual confounding or measurement differences in PA classification. Nonetheless, our findings support that promoting PA among older Saudi adults could contribute to lower T2DM prevalence.

An unhealthy diet was associated with significantly higher T2DM prevalence (24.6% vs. 19.3% for a healthy diet) (χ^2^ = 11.36, *p* = 0.001). This 5.3 percentage point difference is clinically meaningful, though causality cannot be inferred. The high reported consumption of soft drinks (67%) and energy drinks (~30%) in KSA [30] may partially explain this association, though we did not directly assess specific dietary components. Reverse causality is possible: individuals diagnosed with T2DM may adopt healthier diets, attenuating the observed association.

Obesity showed the strongest association with T2DM in our cohort: prevalence was 37.1% among obese participants, compared with 20.8% among overweight and 14.9% among normal-weight individuals (χ^2^ = 136.47, *p* < 0.001). This gradient—a 22.2 percentage point difference between obese and normal-weight groups—is consistent with previous Saudi research demonstrating a strong association between obesity and T2DM [31]. These findings highlight weight management as a priority intervention target in this population.

Smokers had significantly higher T2DM prevalence (26.6%) than non-smokers (21.1%) (χ^2^ = 6.20, *p* = 0.013). This 5.5 percentage point difference aligns with established evidence linking smoking to insulin resistance [32,33]. Given that smoking prevalence in KSA shows marked gender disparity (men: 27.5%; women: 3.7%) [34], the overall association we observed may be driven predominantly by male participants.

### 4.2. Hypertension

In this cohort, the overall prevalence of HTN was 13.4%, which is lower than the nationally reported pooled prevalence of 22.7% [35]. This discrepancy may reflect differences in case definition (self-reported diagnosis vs. measured blood pressure) or age distribution, as undiagnosed HTN is common in Saudi Arabia.

HTN prevalence was significantly associated with age: from 7.2% (age 30–40) to 17.3% (age > 60) (χ^2^ = 35.99, *p* < 0.001). This age-related increase is consistent with previous Saudi reports [36,37].

HTN prevalence was higher in females (14.0%) than males (12.2%), but this difference was not statistically significant (χ^2^ = 1.85, *p* = 0.174). Previous Saudi studies have reported that postmenopausal women have higher rates than men, consistent with a study that reported 13.78% in women vs. 8.5% in men [38,39]. Our finding of a non-significant difference may reflect that a substantial portion of our female participants were premenopausal, or that our sample size was insufficient to detect a small sex difference.

Inactive participants had significantly higher HTN prevalence (14.6%) than active participants (11.1%) (χ^2^ = 7.41, *p* = 0.007). Previous Saudi data reported that 78.2% of hypertensive individuals have insufficient PA [38]. Our findings confirm an inverse association between PA and HTN. However, due to the cross-sectional design, we cannot determine whether inactivity precedes HTN or whether individuals diagnosed with HTN become less active.

An unhealthy diet was associated with significantly higher HTN prevalence (16.3% vs. 10.8% for healthy diet) (χ^2^ = 18.80, *p* < 0.001). This finding aligns with recent national data that indicate poor adherence to dietary guidelines, with one 2025 study reporting an average adherence score of only 26 out of 100 points among Saudi adults [40], and another finding that 56.1% of young Saudi females showed low adherence to national dietary recommendations [41].

Obesity was significantly associated with higher HTN prevalence (17.6% in obese, 16.2% in overweight, 9.3% in normal-weight) (χ^2^ = 33.95, *p* < 0.001). Recent research reported that individuals with Class 3 obesity (BMI ≥ 40 kg/m^2^) had 2.60 times the odds of HTN compared to those with normal BMI [42]. Notably, the increase from normal weight to overweight (9.3% to 16.2%) was steeper than from overweight to obese (16.2% to 17.6%), suggesting that preventing the transition from normal weight to overweight may be particularly important for HTN prevention.

Smokers had higher HTN prevalence (15.3%) than non-smokers (13.1%), but this difference was not statistically significant (χ^2^ = 1.54, *p* = 0.215). Recent research in KSA has confirmed that smokers exhibit higher blood pressure readings, with heavy smokers showing elevated systolic and diastolic levels compared to non-smokers [43]. The non-significant finding in our study may reflect our binary classification of smoking (which does not capture intensity or pack-years) or the low proportion of female smokers in our sample.

### 4.3. Osteoporosis

In this cohort, the overall prevalence of OP was 22.0%, which falls within the range reported in previous Saudi studies (23.4% to 39.5%) and is slightly higher than the global meta-analytic estimate of 18.3% [44].

OP prevalence was significantly associated with age: 7.4% (age 30–40) to 30.3% (age > 60) (χ^2^ = 148.63, *p* < 0.001). This strong age-related association is consistent with the previous literature [45].

In this cohort, OP prevalence was significantly higher in females (23.4%) than in males (19.7%) (χ^2^ = 5.01, *p* = 0.025). This female predominance is consistent with young females being at higher risk for OP due to low dairy consumption and sedentary behavior [41]. Females aged 50 years and older have a four-fold higher prevalence of OP and a two-fold higher prevalence of osteopenia than males [44].

Physically active participants had significantly lower OP prevalence (19.0%) than inactive participants (23.8%) (χ^2^ = 8.92, *p* = 0.003). Previous Saudi research reported that PH is more prevalent among individuals with OP (60.65%) than without (39.32%) [46]. Consistent physical activity can contribute to improved BMD [45]. However, due to the cross-sectional design, we cannot determine whether physical inactivity precedes OP or whether individuals with OP become less active due to pain or frailty.

The difference in OP prevalence between a healthy diet (21.9%) and an unhealthy diet (22.2%) was minimal and not statistically significant (χ^2^ = 0.07, *p* = 0.791). This finding aligns with a recent 2025 meta-analysis, which reported that Western/unhealthful dietary patterns are not significantly associated with OP risk (OR: 1.12; 95% CI: 0.78–1.62; *p* > 0.05) [47].

No significant association was observed between body weight and OP prevalence: 21.8% in normal-weight, 22.0% in overweight, and 22.5% in obese individuals (χ^2^ = 0.20, *p* = 0.905). This finding contrasts with international studies reporting a protective effect of higher BMI against OP, where excess body weight places mechanical stress on the skeleton—thereby stimulating osteoblast activity and increasing BMD—reinforcing BMI as a protective determinant of bone mass [48]. The absence of an association in our cohort may reflect that our participants were predominantly young to middle-aged adults, or that the mechanical and hormonal effects of obesity on bone density are more closely linked to fracture risk than to the diagnosis of OP [49].

Smokers had higher OP prevalence (24.3%) than non-smokers (21.7%), but this difference was not statistically significant (χ^2^ = 1.37, *p* = 0.242). This non-significant finding may reflect our binary classification of smoking (which does not capture pack-years or duration) or the low proportion of female smokers in our sample. However, cigarette smoking is a major lifestyle risk factor for bone health, as it triggers cellular and molecular disruptions that cause an imbalance in bone turnover, ultimately leading to reduced bone mass, osteoporosis, and an increased risk of fractures [50].

### 4.4. Chronic Obstructive Pulmonary Disease (COPD)

In this cohort, the overall prevalence of respiratory conditions was 4.3%, which aligns closely with the nationally reported prevalence of 4.2% for COPD among Saudi adults aged 40 years and older [51].

Respiratory condition prevalence increased with age from 2.6% (age 30–40) to 5.4% (age > 60), but this association was not statistically significant (χ^2^ = 7.47, *p* = 0.058, borderline). This age-related pattern is consistent with previous Saudi reports on COPD prevalence [52].

Prevalence was higher in males (5.1%) than females (3.8%), but this difference was not statistically significant (χ^2^ = 3.78, *p* = 0.052, borderline). This observation concurs with earlier Saudi studies, which have determined that the prevalence of COPD in the population aged 40 years or older is greater in males (5.7%) than in females (2.5%) [51].

Physically active participants had lower respiratory condition prevalence (3.4%) than inactive participants (4.8%), but this difference was not statistically significant (χ^2^ = 2.43, *p* = 0.119). However, a 2026 study reported an inverse relationship between PA and COPD prevalence, finding that inactive adults had a significantly higher prevalence (9.15%) than active adults (3.50%) [53].

Participants with healthy diets had significantly lower respiratory condition prevalence (3.2%) than those with unhealthy diets (5.5%) (χ^2^ = 9.55, *p* = 0.002). This finding aligns with prior evidence that suggests that adherence to a healthy dietary pattern can reduce the risk of lung impairment, while a Western-style (unhealthy) diet is associated with a higher risk of COPD [54].

Obesity was significantly associated with higher respiratory condition prevalence: 2.4% in normal-weight, 5.2% in overweight, and 6.7% in obese individuals (χ^2^ = 21.42, *p* < 0.001). A Saudi study reported that 71% of patients with COPD were either overweight or obese [55]. Our findings confirm a strong association between obesity and respiratory conditions in this population.

Smoking was significantly associated with higher respiratory condition prevalence: 7.9% in smokers vs. 3.7% in non-smokers (χ^2^ = 19.57, *p* < 0.001). This finding is in line with a substantial body of evidence in which the persistent oxidative stress triggered by long-term cigarette smoke exposure is implicated in the development of COPD [14], including in Saudi Arabia, where COPD prevalence rose by 48.6% from 1990 to 2019, with smoking driving 44% of the disease burden, reinforcing the link between smoking and rising COPD rates [56].

### 4.5. Chronic Kidney Disease (CKD)

In this cohort, the overall prevalence of renal disease was 5.1%, which is lower than the global estimate of approximately 10% [57]. This discrepancy may reflect that our renal disease variable captures diagnosed CKD only, whereas many individuals with early-stage CKD remain undiagnosed.

Renal disease prevalence increased significantly with age: from 2.4% (age 30–40) to 7.1% (age >60) (χ^2^ = 12.31, *p* = 0.006). The age-related rise in CKD is largely driven by the increasing prevalence of key risk factors such as diabetes, HTN, obesity, and CVD [58].

Prevalence was slightly higher in females (5.2%) than males (4.9%), but this difference was not statistically significant (χ^2^ = 0.11, *p* = 0.740). This finding aligns with a previous study that reported no significant sex-based differences with respect to disease duration or associated risk factors. However, compared with females, males have a 50% greater likelihood of disease progression to end-stage renal disease (ESRD) [58].

Inactive participants had higher renal disease prevalence (5.4%) than active participants (4.6%), but this difference was not statistically significant (χ^2^ = 0.63, *p* = 0.427). In contrast, a higher level of moderate-to-vigorous PA demonstrated an inverse association with CKD risk, corresponding to a 38% lower likelihood of developing the condition [59]. Similarly, relative to light PA, moderate and vigorous activity lowered the risk of incident CKD by 13% and 21%, respectively [60].

Participants with unhealthy diets had higher renal disease prevalence (5.4%) than those with healthy diets (4.8%), but this difference was not statistically significant (χ^2^ = 0.13, *p* = 0.718). Nevertheless, despite this null finding in our cohort, dietary modification is widely recognized as a key preventive measure and a cornerstone of therapeutic intervention for CKD [60]. A recent meta-analysis, comprising six studies with a total of 568,213 participants and 16,694 documented CKD cases, demonstrated a statistically significant inverse relationship between adherence to high-quality dietary patterns, specifically the Dietary Approaches to Stop HTN, and the risk of developing CKD [61].

Renal disease prevalence associated with body weight (4.3% in normal-weight, 5.3% in overweight, 6.4% in obese), but this association was not statistically significant (χ^2^ = 3.89, *p* = 0.143). However, our findings align with a 2024 Saudi registry study of 2912 CKD patients, which reported that 37% were obese and 25% were overweight—reinforcing the well-established association between obesity and CKD, wherein obesity triggers glomerular and tubular injury via inflammatory pathways, despite the non-significant association observed in our cohort [62].

Smokers had significantly higher renal disease prevalence (7.1%) than non-smokers (4.8%) (χ^2^ = 4.57, *p* = 0.033). This finding aligns with previous research showing that active smoking is an independent risk factor for both the development of CKD and its accelerated progression to ESKD. The toxic effects of smoking are well documented and involve both direct oxidative stress-mediated nephrotoxicity and indirect renal injury [63]. Consistent with this, a Saudi study reported that 33.3% of the CKD population are smokers [62].

### 4.6. Strengths and Limitations

There are several limitations to this study. While the cross-sectional design precludes causal inference, it provides a practical approach to estimate disease prevalence and identify associated factors. The large sample size (*n* = 2877) and simultaneous assessment of multiple NCDs and lifestyle factors are significant strengths.

Although self-reported data may introduce bias, this approach enabled efficient data collection using validated instruments (IPAQ, GATS) and remains the most practical method for assessing multiple lifestyle behaviors in population-based studies.

Although convenience sampling via online platforms limits generalizability, it enabled efficient recruitment from multiple Saudi provinces. The consistency of our prevalence estimates with national data (e.g., T2DM: 21.8% vs. 23.7%) suggests reasonable representativeness despite sampling limitations.

Binary classification of lifestyle factors has limitations, including loss of dose-response information. However, this approach aligns with WHO guidelines, enables comparison with surveillance data, and identifies intervention targets. All cutoffs were based on validated instruments (IPAQ, AHEI, GATS) and recognized standards.

Chi-square tests were appropriate for our categorical variables, allowing efficient testing of associations across 30 comparisons. The large sample size ensured stable estimates, while Microsoft Excel ensured transparency and reproducibility.

A major limitation of this study is the absence of multivariate logistic regression analysis. Our chi-square tests examined unadjusted associations only and did not account for potential confounding factors such as age, sex, and the overlap between lifestyle factors (e.g., smoking is more prevalent in men; obesity increases with age). Therefore, we cannot determine whether the observed associations are independent or driven by confounders. We recommend that future studies employ multivariate logistic regression to confirm the independent associations reported here.

## 5. Conclusions

In this cross-sectional study of 2877 Saudi adults, lifestyle factors were associated with both WHO core NCDs (T2DM, HTN, respiratory conditions) and NCD-associated conditions (OP, CKD). Obesity showed the strongest association with T2DM (37.1% in obese vs. 14.9% in normal-weight participants), while smoking was most strongly associated with respiratory conditions (7.9% in smokers vs. 3.7% in non-smokers). Unhealthy diet and physical inactivity were also significantly associated with multiple NCDs. Sex differences were significant only for OP (female predominance). Due to the cross-sectional design, causal inferences cannot be drawn. Nevertheless, these findings underscore an urgent need for targeted public health interventions to promote PA, improve nutrition, combat obesity, and reduce smoking.

## Figures and Tables

**Table 1:** Lifestyle profiles of the study participants.

Item	Variable	No.	Percentage
Physical activity	Active individual	1044	36.3%
	Inactive individual	1833	63.7%
Smoking	Non-smokers	2385	82.9%
	Smokers	492	17.1%
	Normal weight	1321	45.9%
Obesity	Overweight	903	31.4%
	Obese	653	22.7%
	Healthy diet	1533	53.3%
Diet	Unhealthy diet	1344	46.7%

**Table 2:** Classification of WHO core non-communicable diseases (NCDs) and NCD-associated conditions.

Category	Diseases
WHO core NCDs	T2DM, COPD, HTN
NCD-associated conditions	OP, CKD

**Table 3:** Variation in age-related disease prevalence by age, sex, and lifestyle factors.

Item	Variable	T2DM	HTN	OP	COPD	CKD
The overall prevalence		627 (21.8%)	384 (13.4%)	634 (22%)	123 (4.3%)	147 (5.1%)
Age	30–40	93 (18.5%)	36 (7.2%)	37 (7.4%)	13 (2.6%)	12 (2.4%)
	41–50	176 (20.8%)	97 (11.4%)	158 (18.6%)	32 (3.8%)	41 (4.8%)
	51–60	209 (23.8%)	139 (15.8%)	243 (27.6%)	43 (4.9%)	48 (5.5%)
	<60	149 (23.0%)	112 (17.3%)	196 (30.3%)	35 (5.4%)	46 (7.1%)
Sex	Male	221 (20.9%)	129 (12.2%)	208 (19.7%)	54 (5.1%)	52 (4.9%)
	Female	406 (22.3%)	255 (14.0%)	426 (23.4%)	69 (3.8%)	95 (5.2%)
PA	Active	196 (18.8%)	116 (11.1%)	198 (19.0%)	35 (3.4%)	48 (4.6%)
	Inactive	431 (23.5%)	268 (14.6%)	436 (23.8%)	88 (4.8%)	99 (5.4%)
Diet	Healthy diet	296 (19.3%)	166 (10.8%)	336 (21.9%)	49 (3.2%)	74 (4.8%)
	Unhealthy diet	331 (24.6%)	219 (16.3%)	298 (22.2%)	74 (5.5%)	73 (5.4%)
Obesity	Normal	197 (14.9%)	123 (9.3%)	288 (21.8%)	32 (2.4%)	57 (4.3%)
	Over weight	188 (20.8%)	146 (16.2%)	199 (22.0%)	47 (5.2%)	47 (5.3%)
	Obese	242 (37.1%)	115 (17.6%)	147 (22.5%)	44 (6.7%)	42 (6.4%)
Smoking	Non-smoker	526 (21.1%)	326 (13.1%)	542 (21.7%)	93 (3.7%)	120 (4.8%)
	Smoker	101 (26.6%)	58 (15.3%)	92 (24.3%)	30 (7.9%)	27 (7.1%)

Abbreviations: PA, physical activity; WHO core NCDs: T2DM, type 2 diabetes; COPD, chronic obstructive pulmonary disease; HTN, hypertension (cardiovascular diseases group). NCD-associated conditions: CKD, chronic kidney disease; OP, osteoporosis.

**Table 4:** Association between lifestyle factors, age groups, and NCDs (chi-square tests).

Lifestyle Factor	Disease	χ^2^	df	*p*-Value
PA	T2DM	8.81	1	0.003
	HTN	7.41	1	0.007
	OP	8.92	1	0.003
	COPD	2.43	1	0.119
	CKD	0.63	1	0.427
Diet	T2DM	11.36	1	0.001
	HTN	18.8	1	<0.001
	OP	0.07	1	0.791
	COPD	9.55	1	0.002
	CKD	0.13	1	0.718
Smoking	T2DM	6.2	1	0.013
	HTN	1.54	1	0.215
	OP	1.37	1	0.242
	COPD	19.57	1	<0.001
	CKD	4.57	1	0.033
Obesity	T2DM	136.47	2	<0.001
	HTN	33.95	2	<0.001
	OP	0.2	2	0.905
	COPD	21.42	2	<0.001
	CKD	3.89	2	0.143
Age Groups	T2DM	8.47	3	0.037
	HTN	35.99	3	<0.001
	OP	148.63	3	<0.001
	COPD	7.47	3	0.058
	CKD	12.31	3	0.006
Sex	T2DM	0.79	1	0.374
	HTN	1.85	1	0.174
	OP	5.01	1	0.025
	COPD	3.78	1	0.052
	CKD	0.11	1	0.740

Note: df = degrees of freedom. Statistical significance set at *p* < 0.05. Abbreviations: PA, physical activity; WHO core NCDs: T2DM, type 2 diabetes; COPD, chronic obstructive pulmonary disease; HTN, hypertension (cardiovascular diseases group). NCD-associated conditions: CKD, chronic kidney disease; OP, osteoporosis.

## Data Availability

All data generated or analyzed during this study are included in this published article.

## Abbreviations

The following abbreviations are used in this manuscript:

PA	Physical activity
NCD	Non-communicable disease
WHO	World Health Organization
T2DM	Type 2 diabetes mellitus
HTN	Hypertension
COPD	Chronic obstructive pulmonary disease
OP	Osteoporosis
CKD	Chronic kidney disease
AHEI	Alternative Healthy Eating Index
WHO GATS	WHO Global Adult Tobacco Survey
KSA	Kingdom of Saudi Arabia
CVD	Cardiovascular diseases
IPAQ	International PA Questionnaire
BMD	Bone mineral density
